# Protective Effect of Chlorogenic Acid and Its Analogues on Lead-Induced Developmental Neurotoxicity Through Modulating Oxidative Stress and Autophagy

**DOI:** 10.3389/fmolb.2021.655549

**Published:** 2021-06-11

**Authors:** Xiuna Ji, Baokun Wang, Yam Nath Paudel, Zhihui Li, Shanshan Zhang, Lei Mou, Kechun Liu, Meng Jin

**Affiliations:** ^1^ Biology Institute, Qilu University of Technology (Shandong Academy of Sciences), Jinan, China; ^2^ Engineering Research Center of Zebrafish Models for Human Diseases and Drug Screening of Shandong Province, Jinan, China; ^3^ Neuropharmacology Research Strength, Jeffrey Cheah School of Medicine and Health Sciences, Monash University Malaysia, Bandar Sunway, Selangor, Malaysia; ^4^ School of Bioengineering, Qilu University of Technology (Shandong Academy of Sciences), Jinan, China

**Keywords:** lead, chlorogenic acid, neochlorogenic acid, cryptochlorogenic acid, zebrafish

## Abstract

Lead (Pb) is among the deleterious heavy metal and has caused global health concerns due to its tendency to cause a detrimental effect on the development of the central nervous system (CNS). Despite being a serious health concern, treatment of Pb poisoning is not yet available, reflecting the pressing need for compounds that can relieve Pb-induced toxicity, especially neurotoxicity. In the quest of exploring protective strategies against Pb-induced developmental neurotoxicity, compounds from natural resources have gained increased attention. Chlorogenic acid (CGA) and its analogues neochlorogenic acid (NCGA) and cryptochlorogenic acid (CCGA) are the important phenolic compounds widely distributed in plants. Herein, utilizing zebrafish as a model organism, we modeled Pb-induced developmental neurotoxicity and investigated the protective effect of CGA, NCGA, and CCGA co-treatment. In zebrafish, Pb exposure (1,000 μg/L) for 5 days causes developmental malformation, loss of dopaminergic (DA) neurons, and brain vasculature, as well as disrupted neuron differentiation in the CNS. Additionally, Pb-treated zebrafish exhibited abnormal locomotion. Notably, co-treatment with CGA (100 µM), NCGA (100 µM), and CCGA (50 µM) alleviated these developmental malformation and neurotoxicity induced by Pb. Further underlying mechanism investigation revealed that these dietary phenolic acid compounds may ameliorate Pb-induced oxidative stress and autophagy in zebrafish, therefore protecting against Pb-induced developmental neurotoxicity. In general, our study indicates that CGA, NCGA, and CCGA could be promising agents for treating neurotoxicity induced by Pb, and CCGA shows the strongest detoxifying activity.

## Introduction

Lead (Pb) is a heavy metal present in the environment—in water, soil, dust, and food which are grown on soils with high Pb content (D’souza et al., 2011; Jomova and Valko, 2011; Bustamante and Macias-Konstantopoulos, 2016). Pb is widely used in the building industry due to its unusual physical–chemical properties (Brannvall et al., 1999; Sanders et al., 2009). As Pb is a persistent metal, it is toxic to humans and animals, especially children (Van Vonderen et al., 2000; Kumar and Scott Clark, 2009). Despite the significant efforts by the governments worldwide to reduce Pb pollution, humans are being constantly exposed to it (Kim H.-C. et al., 2015). Pb-induced health risk, mainly exhibited as damage to the CNS, has been reported as the major public health issue (Mason et al., 2014). Many studies have shown that exposure to even a small amount of Pb causes changes in the way the body functions (Mahaffey, 1991; Spriewald et al., 1999; Soni and Dayal, 2019). For instance, 10 μg/dl (equivalent to 0.48 μmol/L) or higher of Pb in the blood are considered toxic and result in neurological disorders, cognitive impairments, hypertension, and other disorders (Patrick, 2006b).

Pb damages cellular components *via* elevated levels of oxidative stress since it directly interrupts the activity of enzymes and deactivates antioxidant sulfhydryl pools (Patrick, 2006a). Furthermore, it is reported that Pb can increase the level of caspase-3 protein expression in the testis of rats (Elgawish and Abdelrazek, 2014), and cause lipid peroxidation of cells and reduction in activities of antioxidant enzyme (Sandhir et al., 1994). Therefore, an organism exposed to Pb has signficantly enhanced oxidative stress, which may inhibit the formation of reactive oxygen species (ROS) to prevent the body from being damaged. The direct neurotoxic actions of Pb include apoptosis, excitotoxicity, impact on neurotransmitter storage, and its release phenomena, mitochondria, second messengers, cerebrovascular endothelial cells, and both astroglia and oligodendroglia (Lidsky and Schneider, 2003).

Phenolic compounds have long been studied for their antioxidant properties because they can scavenge harmful ROS (Sanchez-Rangel et al., 2014). Chlorogenic acid (CGA) is the abundantly available biologically active polyphenolic constituent of coffee, possessing arrays of biological activities including antioxidant, antibacterial, hepato- and cardioprotective, hypoglycemic, anti-obesity, anti-inflammatory, and anticarcinogenic effects (Tajik et al., 2017; Naveed et al., 2018). An earlier study reported that CGA can ameliorate Pb-induced renal toxicity (Zhang T. et al., 2019) and arsenite toxicity (Metwally et al., 2020).

CGA and its analogues neochlorogenic acid (NCGA) and cryptochlorogenic acid (CCGA) are the important phenolic compounds that are widely distributed in plants (Mei et al., 2020). NCGA is a natural polyphenolic compound found in dried fruits and other plants (Kim M. et al., 2015). NCGA has exerted protective effect against lipopolysaccharide (LPS)-induced inflammatory response *via* upregulation of Nrf2/HO-1 and involving the AMPK pathway (Park et al., 2018). CCGA is a special isomer of CGA, and the pharmacological effects and related molecular mechanisms of CCGA have been poorly reported (Zhao et al., 2020). An earlier study has reported that CCGA exerts its effect against LPS-induced oxidative stress and inflammation (Zhao et al., 2020).

In the last few decades, zebrafish have gained increased attention as a model organism to study the pathogenesis of human diseases (Lieschke and Currie, 2007), drug discovery (Zon and Peterson, 2005; MacRae and Peterson, 2015), and toxicity studies (Liu et al., 2012; Bhagat et al., 2020). The advantages of small size, economic maintenance, ease of breeding, short life cycle, and high fertility make zebrafish preferable over the rodent model system. Talking precisely on the toxicity, zebrafish have been utilized as a model organism to study neurotoxicity caused by nanoparticles (Jin et al., 2021) and developmental neurotoxicity caused by Pb (Liu et al., 2019), tris-phosphate (Li et al., 2018), boscalid (Wang et al., 2020), and bisphenol F (Gu et al., 2020). Zebrafish embryos/larvae have emerged as an ideal model for evaluating developmental neurotoxicity related to toxicant exposure (Scholz et al., 2008).

To the best of our knowledge, no study has earlier reported the protective effect of CGA, NCGA, and CCGA on Pb-induced developmental neurotoxicity in zebrafish. The exploration and development of effective interventions that can effectively reduce oxidative stress and autophagy are crucial to prevent Pb-induced neurotoxicity. Hence, we aimed to investigate the protective effect upon co-treatment with CGA and its analogues, NCGA and CCGA, on Pb-induced developmental neurotoxicity, and unravel its underlying mechanism in the zebrafish model. The findings from the current study will increase our knowledge regarding the protective effect of CGA, NCGA, and CCGA against Pb-induced developmental toxicity and the underlying mechanisms, which support the use of this novel candidate against Pb-induced toxicity.

## Materials and Methods

### Chemical

Lead acetate (ACS no. 6080–56–4) was purchased from Sigma-Aldrich. CGA, NCGA, and CCGA were purchased from Shanghai Yuanye Bio-Technology Co., Ltd. The Pb stock solutions were prepared in double distilled water (ddH_2_O), and serial dilutions were made in the normal bathing medium. All other chemicals and reagents utilized in this study were of analytical grade.

### Animals and Maintenance

All the experimental procedures were carried out as per the NIH Guide for the Care and Use of Laboratory Animals. Experimental protocols and procedures were approved by the Animal Committee of Biology Institute of Shandong Academy of Sciences and were in accordance with the guideline for the Care and Use of Laboratory Animals of China. Adult zebrafish were raised separately according to their gender under a 14/10 h of light/darkness cycle photoperiod. Zebrafish (*Danio rerio*) of the wild-type AB, *vmat2:GFP*, *elavl3:EGFP*, and *vegfr2:GFP* strains were maintained according to standard procedures (Westerfield, 2000). Fish were kept under a 14-h light/10 h dark cycle photoperiod and fed twice a day with commercial flake fish food supplemented with live brine shrimp. Zebrafish embryos were obtained from the natural mating of adult zebrafish bred and maintained in an automated recirculating system with charcoal-filtered tap water. Fertilized eggs were collected, washed, and transferred to sterile cell culture plates. Plates were maintained in an incubator at 28 ± 0.5°C.

### Drug Treatments and Pb-Induced Developmental Neurotoxicity

Developmental neurotoxicity was induced in zebrafish larvae at 4 hpf *via* exposure to 1,000 μg/L Pb. Co-treatment with CGA, NCGA, and CCGA was performed to investigate the protective effect of CGA, NCGA, and CCGA against Pb-induced neurodevelopmental toxicity in zebrafish larvae. Change in the medium was performed once every 24 h when any dead embryos were discarded. All the experimental animals were divided into 5 corresponding experimental groups, as shown in Table 1. Three replicates were run for each group, each with *n* = 15 animals per group. The concentrations of CGA (100 µM), NCGA (100 µM), and CCGA (50 µM) were selected based on the preliminary studies from our laboratory.

**TABLE 1:** Experimental groups evaluating the protective effect of CGA, CCGA, and NGA against Pb-induced neurodevelopmental toxicity in zebrafish.

Experimental groups	Group code	Description
Group 1	Ctl	Vehicle (0.5% DMSO)
Group 2	Pb	1000 μg/L Pb
Group 3	Pb + CGA	CGA (100 µM) co-treatment + Pb (1,000 μg/L)
Group 4	Pb + NCGA	NCGA (100 µM) co-treatment + Pb (1,000 μg/L)
Group 5	Pb + CCGA	CCGA (50 µM) co-treatment + Pb (1,000 μg/L)

### Assessment of Developmental Neurotoxicity

Developmental neurotoxicity induced by Pb was assessed as per our earlier reported study (Jin et al., 2019b; Shang et al., 2020). The mortality rate evaluated from 24 to 120 hpf was identified by coagulation of the larvae. We recorded embryo morphology at 120 hpf and analyzed the malformation rate, whereas the hatching rate was examined at 48 and 72 hpf. The tests were repeated three times, each with *n* = 15 zebrafish per group.

### Toxicity Scoring

Toxicity scores were assessed at 120 hpf as per the earlier reported studies (Bar-Ilan et al., 2009; Liu et al., 2012). The toxicity score ranges from 0 to 4, where 0 denotes normal development, 1 denotes one or two minor malformations, 2 denotes one moderate or three to four minor malformations, 3 indicates more than one severe or four minor malformations, and 4 indicates embryonic lethality (Bar-Ilan et al., 2009). Several types of malformations were considered in the current investigation, and these include pericardial edema (PE), yolk sac malformation (YM), bent spine (BS), small eyes (SE), tail malformation (TM), Meckel's cartilage hypoplasia (MH), and absence of a swimming bladder (AB). The toxicity scores and types of malformation were visualized and recorded by a stereomicroscope (OLYMOPUS SZX 10, Japan) and were documented photographically. The scoring was repeated 3 times, each with n = 6 animals per group.

### Evaluation of Regions of Dopaminergic Neurons, Differentiated Central Nervous System Neurons, and Blood Vessels in the Brain

Zebrafish strains *vmat2:GFP*, *elavl3:EGFP*, and *fli1:GFP* were used in these assays. After treatment, we randomly selected 6 individuals per group to observe phenotypes and acquire images. Then we measured the length of DA neurons, and the integrated optical density (IOD) of the head and notochord (which had been marked by red dotted line) using Image-Pro Plus software (Media Cybernetics, Bethesda, United States) to evaluate the changes of DA neurons and differentiated CNS neurons, respectively. We also counted the number of blood vessels in the brain to evaluate the loss of vasculature.

### Locomotor Activity Test

Treated zebrafish larvae were transferred into 48-well plates at 120 hpf (one larva per well), and then left in aquarium water (500 μL per well) for acclimatization. 30 min post-acclimatization, the locomotion activities of each larva from different experimental groups were recorded immediately by Zeblab video-tracking system (Viewpoint, Lyon, France) for 20 min. Digital tracks and average speed were analyzed every minute. The tests were repeated three times, each with *n* = 10 zebrafish per group.

### Real-Time Quantitative PCR

The real-time qPCR was performed to investigate the modulation in mRNA levels of *c-fos*, *gfap*, *mbp*, *pparγ*, *tuba1b*, *bdnf*, *dat*, *cat*, *sod2*, *sod1*, *cat*, *gclm*, *gsto2*, *gpx41*, *dj1*, *pink1*, *parkin*, *ambra1a*, *ulk1b*, *ulk2*, and *atg5.* Larval zebrafish from each treatment group were extracted using EASY spin Plus RNA Mini Kit (Aidlab Biotechnologies; Beijing, China). Extracted RNA was then reverse-transcribed into cDNA using the PrimeScript™ RT Master Mix (Takara; Tokyo, Japan). The real-time qPCR was performed using SYBR^®^ Premix DimerEraser™ (Takara, Tokyo, Japan) and Light Cycler^®^ 96 System (Light Cycler^®^ Instrument; Roche; Switzerland). Runs were carried out in triplicate, and then normalized to housekeeping gene rpl13a to evaluate the mRNA level of genes of interest. Data were analyzed using LC 96 Application Software and then calculated according to the 2−ΔΔCt method to quantify the relative gene expression (Livak and Schmittgen, 2001). The primers used in this study were listed in Supplementary Table S1. Three replicates were run for each group, each with *n* = 20–30 animals per group.

### Statistical Analysis

Data were analyzed using Graph Pad Prism 8.0 (GraphPad Software; CA, United States) *via* one-way ANOVA followed by Dunnett’s multiple comparison test and were presented as mean ± SEM. *p* < 0.05 was considered as significant.

## Results

### Effect of Chlorogenic Acid, Neochlorogenic Acid, and Cryptochlorogenic Acid on Zebrafish Morphology, Hatching Rate, and Mortality Rate

**FIGURE 1:**
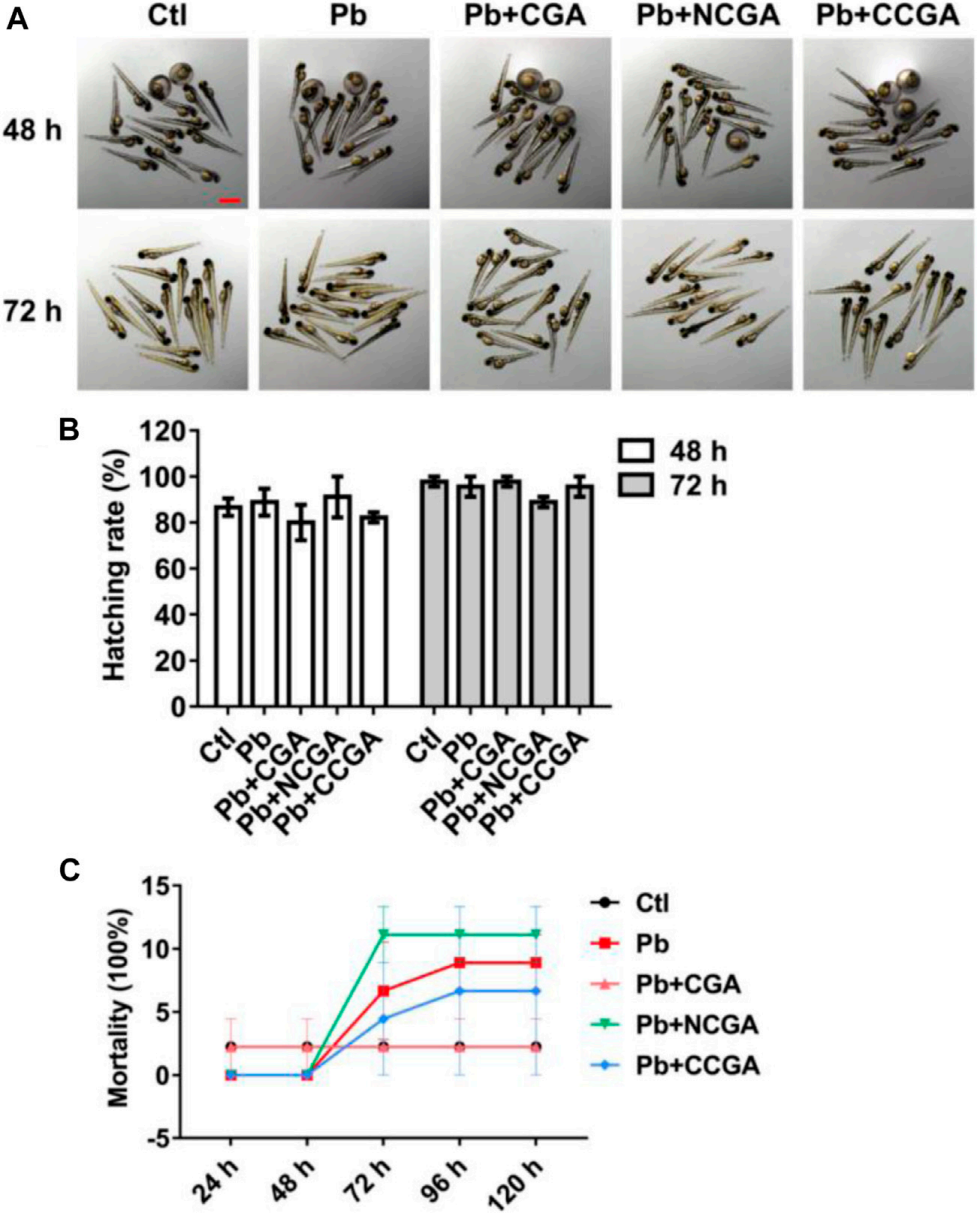
Hatching and mortality rate of zebrafish larvae. **(A)** Representative images of the morphology of zebrafish exposed to different groups at 48 and 72 hpf (scale bar, 1 mm). **(B)** Hatching rates at 48 and 72 hpf. **(C)** Mortality rate of zebrafish in different groups from 24 to 120 hpf.

The zebrafish embryos co-treated with CGA, NCGA, and CCGA at 48 and 72 h exhibited normal development as the control group (Figure 1A). The hatching rate was not significantly affected by Pb treatment at 48 and 72 hpf (Figure 1B). Similarly, co-treatment with CGA, NCGA, and CCGA also exerted no significant difference in the hatching rate both at 48 and 72 hpf (Figure 1B).

To assess the toxicity *in vivo*, wild-type zebrafish larvae were examined after treatment with Pb, CGA, NCGA, and CCGA. No 3significant difference in the mortality rate was observed in the Pb-, CGA-, NCGA-, and CCGA-treated groups from 48 h to 120 hpf (Figure 1C).

### Effect on Toxicity Score and Phenotypic Deficits

**FIGURE 2:**
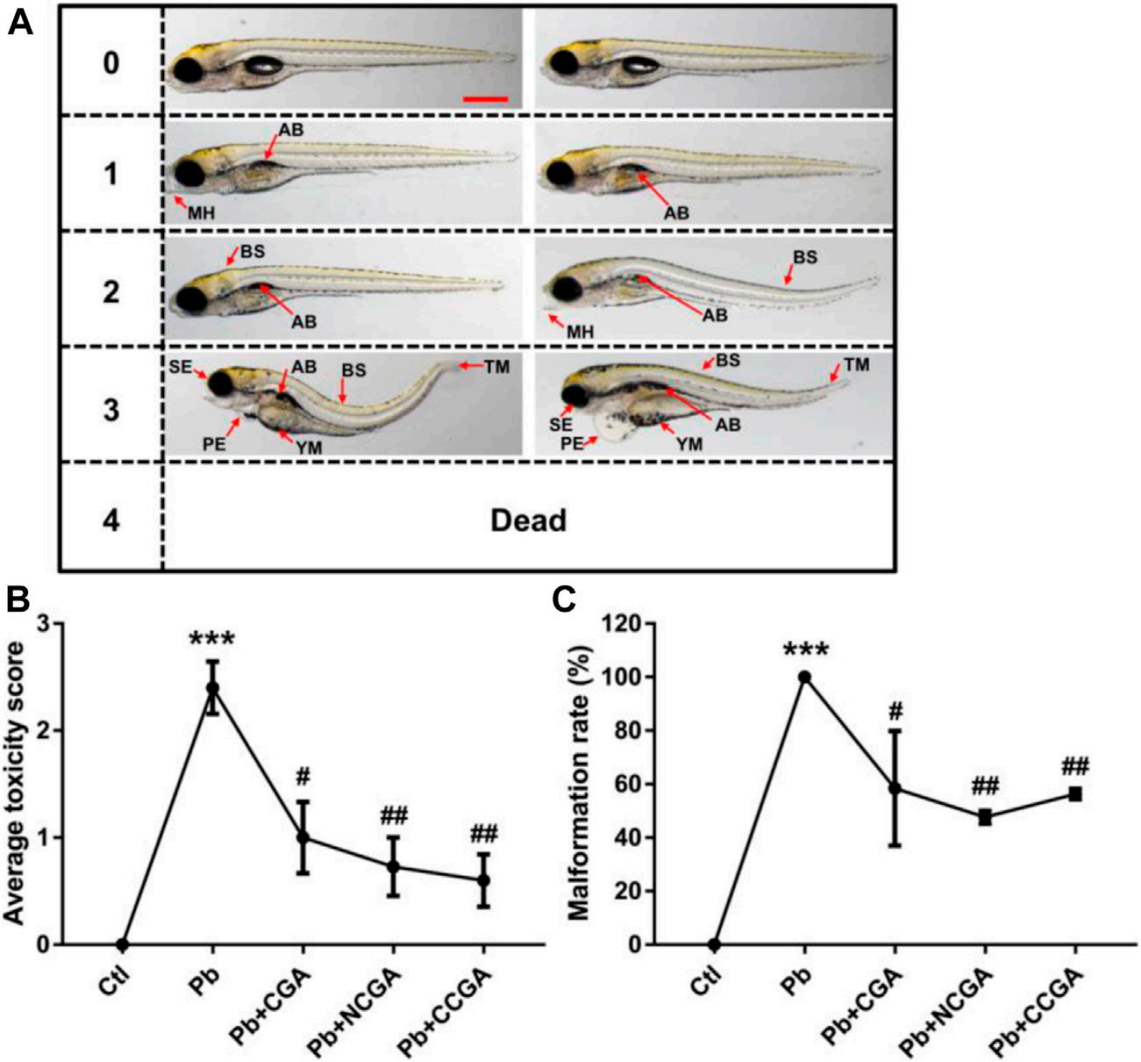
Micrographs, average toxicity scores, and malformation rate. **(A)** Representative images of toxicity score from 0 to 4 at 120 hpf. Zebrafish malformation was scored from level 0 to level 4.0, zebrafish developed normally; 1–3, zebrafish had slight-to-severe defects; 4, embryonic lethality. Two images for each of semiquantitative toxicity scoring indicate that both of these abnormalities are ranked as the same toxicity score (scale bar, 500 μm). **(B)** Average toxicity score at 120 hpf (*n* = 6); ^***^ *p* < 0.001 vs. control and ^#^ *p* < 0.05, ^##^ *p* < 0.01 vs. Pb. **(C)** Pb-induced phenotypic defects and malformation rate (*n* = 15); ^***^ *p* < 0.001 vs. control and ^#^ *p* < 0.05, ^##^ *p* < 0.01 vs. Pb.

Figure 2A demonstrates the scoring spectra at 120 hpf. When compared to the control group, the average toxicity score was significantly increased in the Pb-treated group (***p < 0.001) (Figure 2B). On the contrary, the Pb-induced toxicity score was reduced significantly upon treatment with CGA (^#^ *p* < 0.05), NCGA (^##^ *p* < 0.01), and CCGA (^##^ *p* < 0.01) (Figure 2B).

The malformation rate in the Pb-treated group was significantly upregulated (***p < 0.001) when compared to that in the control group (Figure 2C), reflecting that Pb exposure induced malformation in zebrafish. The Pb-induced upregulation in the malformation rate was, in turn, reduced significantly upon treatment with CGA (^#^ *p* < 0.05), NCGA (^##^ *p* < 0.01), and CCGA (^##^ *p* < 0.01) (Figure 2C).

Our findings reflect that CGA, NCGA, and CCGA can alleviate the malformation of zebrafish caused by Pb.

### Effect on the Length of Dopaminergic Neuron Region

**FIGURE 3:**
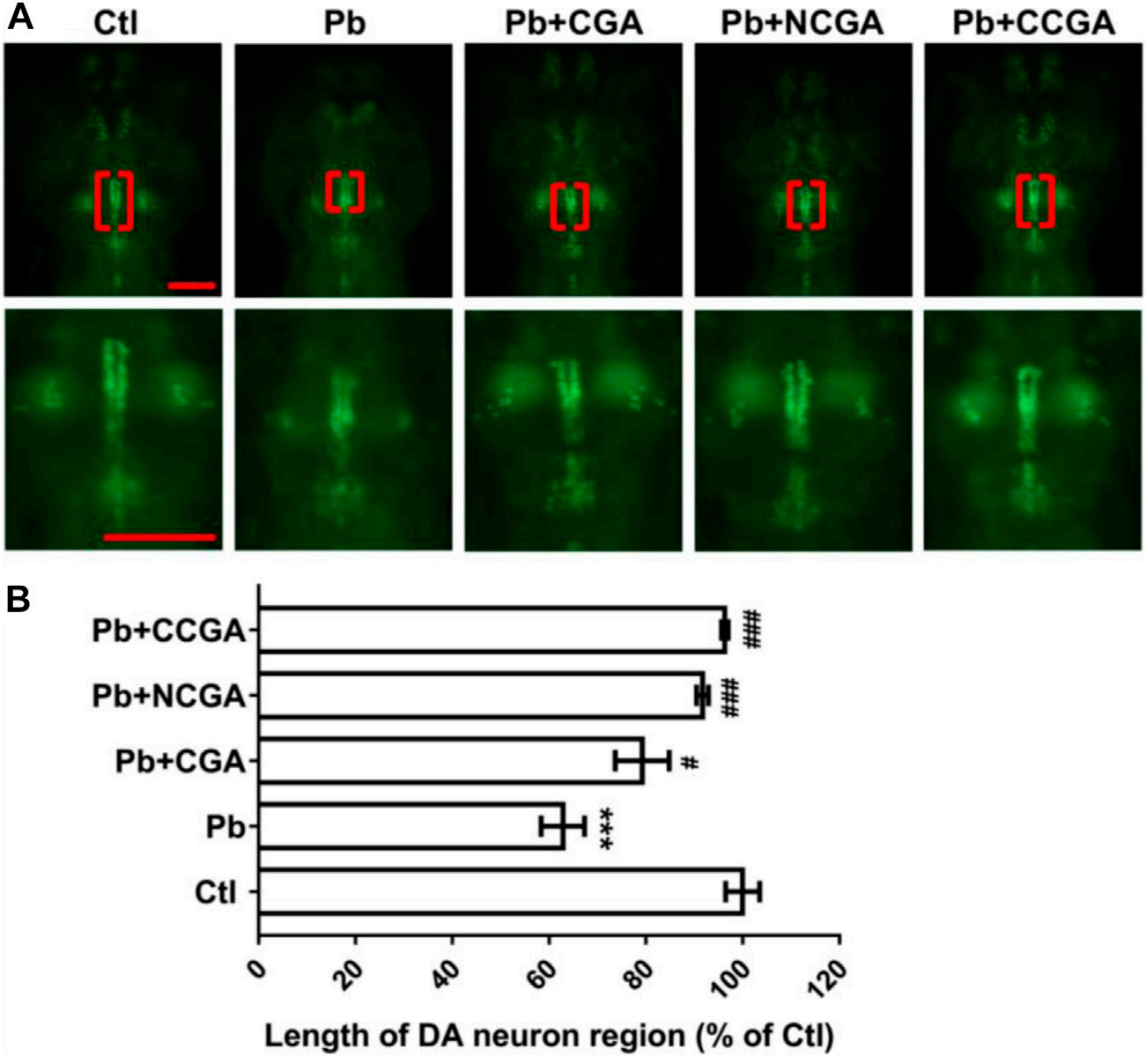
Representative images of DA neuron of zebrafish and its analysis. **(A)** Representative fluorescence microscopy images of vmat2:GFP zebrafish at 120 hpf. DA neurons were indicated by the red brackets. Enlarged images are shown to improve visualization of DA neuron morphology (scale bar, 100 μm). **(B)** Statistical analysis of the length of the DA neuron region (*n* = 6); ^***^ *p* < 0.001 vs. control and ^#^ *p* < 0.05, ^###^ *p* < 0.001 vs. Pb.

In order to assess the impact of Pb-induced apoptosis and the protective effect of CGA, NCGA, and CCGA against the same, we have studied the modulation in the length of DA neurons in zebrafish. At 120 hpf, compared to the control group, there was a significant (^***^ *p* < 0.001) decrease in the length of DA neurons in the Pb-treated group. On the contrary, co-treatment with CGA (^**#**^ *p* < 0.05), NCGA (^**###**^ *p* < 0.001), and CCGA (^**###**^ *p* < 0.001) significantly reduced the Pb-induced DA neuron loss in zebrafish (Figures 3A,B). Our findings suggest that CGA and its analogues NCGA and CCGA can inhibit the loss of DA neurons in zebrafish induced by Pb.

### Chlorogenic Acid, Neochlorogenic Acid, and Cryptochlorogenic Acid Effect on Differentiated Central Nervous System Neuron Region

**FIGURE 4:**
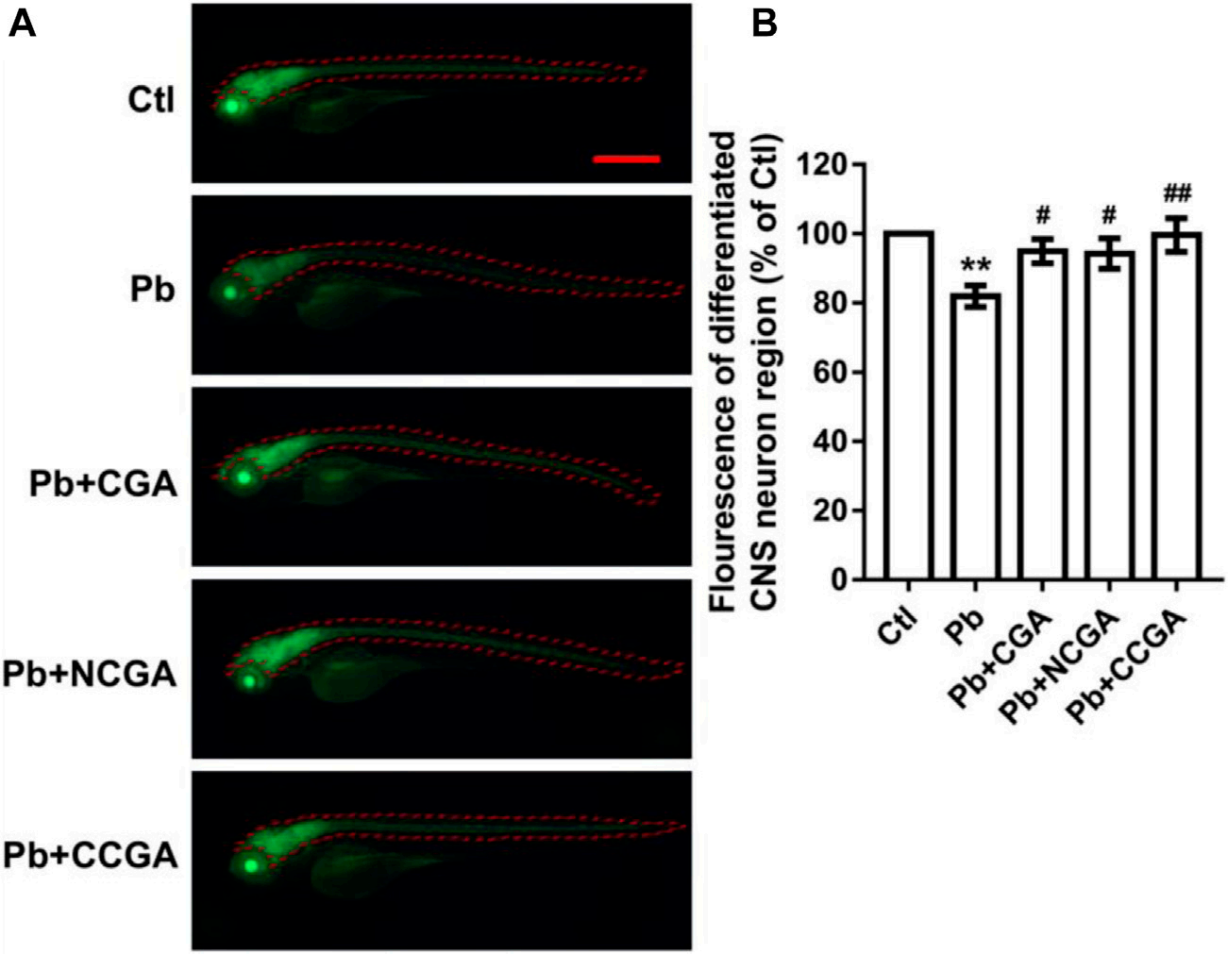
Representative fluorescence images of differentiated CNS neuron region of zebrafish and its analysis. **(A)** Representative fluorescence microscopy images of elavl3:EGFP zebrafish at 120 hpf (scale bar, 500 μm). **(B)** Statistical analysis of the fluorescence of differentiated CNS neuron region (*n* = 6);^**^ *p* < 0.01 vs. control and ^#^ *p* < 0.05, ^##^ *p* < 0.01 vs. Pb.

With an aim to study whether co-treatment with CGA, NCGA, and CCGA protected the neuron differentiation in the CNS, we have analyzed the fluorescence of differentiated CNS neuron region. We observed a significant reduction (**p < 0.01) in the fluorescence of differentiated CNS neuron region in the Pb-treated group when compared to the control group. However, when compared to the Pb-treated group, there was a significant upregulation in the fluorescence of differentiated CNS neuron region upon co-treatment with CGA (^**#**^ *p* < 0.05), NCGA (^**#**^ *p* < 0.05), and CCGA (^##^ *p* < 0.01) (Figures 4A,B).

### Effect on the Number of the Blood Vessel in the Brain

**FIGURE 5:**
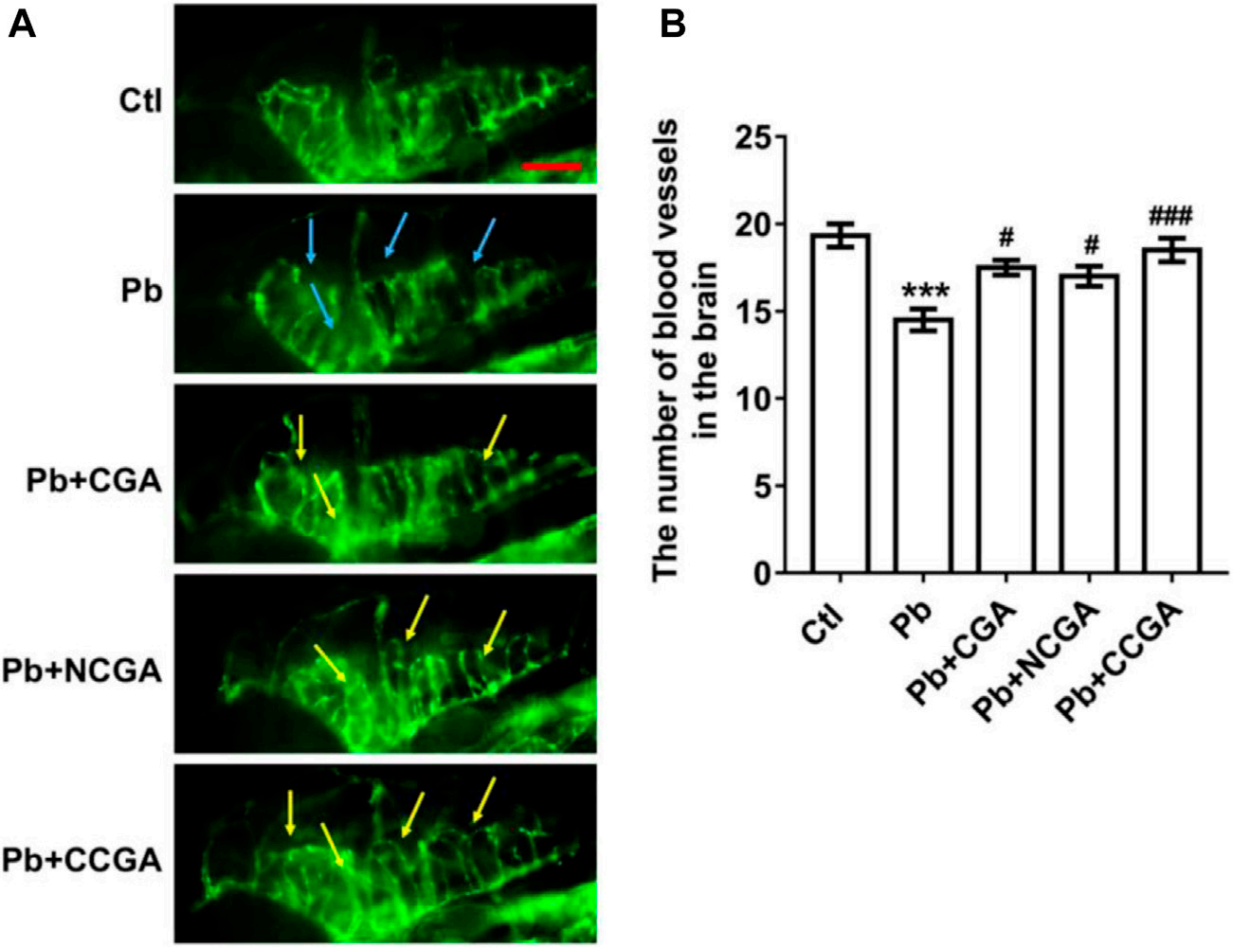
Representative images of blood vasculature of zebrafish and its analysis. **(A)** Representative fluorescence microscopy images of fli1:GFP zebrafish at 120 hpf. Loss of vasculature was indicated by blue arrows. Recovery of vasculature was indicated by yellow arrows. (scale bar, 100 μm). **(B)** Statistical analysis of the number of blood vessels in the brain (*n* = 6). ^***^ *p* < 0.001 vs. control and ^#^ *p* < 0.05, ^###^ *p* < 0.001 vs. Pb.

To assess the loss of vasculature in zebrafish after Pb administration and the protective effect of CGA, NCGA, and CCGA co-treatment against the same, we have detected the number of blood vessels in the brain. We observed that Pb administration leads to a significant (^***^ *p* < 0.001) decline in the number of blood vessels in the brain when compared to the control group. The Pb-induced reduction in the number of blood vessels in the brain were significantly ameliorated upon treatment with CGA (^**#**^ *p* < 0.05), NCGA (^**#**^ *p* < 0.05), and CCGA (^###^ *p* < 0.001) (Figures 5A,B). Our finding reflects that CGA, NCGA, and CCGA can inhibit the loss of vasculature in zebrafish induced by Pb.

### Effect in Locomotor Activity

**FIGURE 6:**
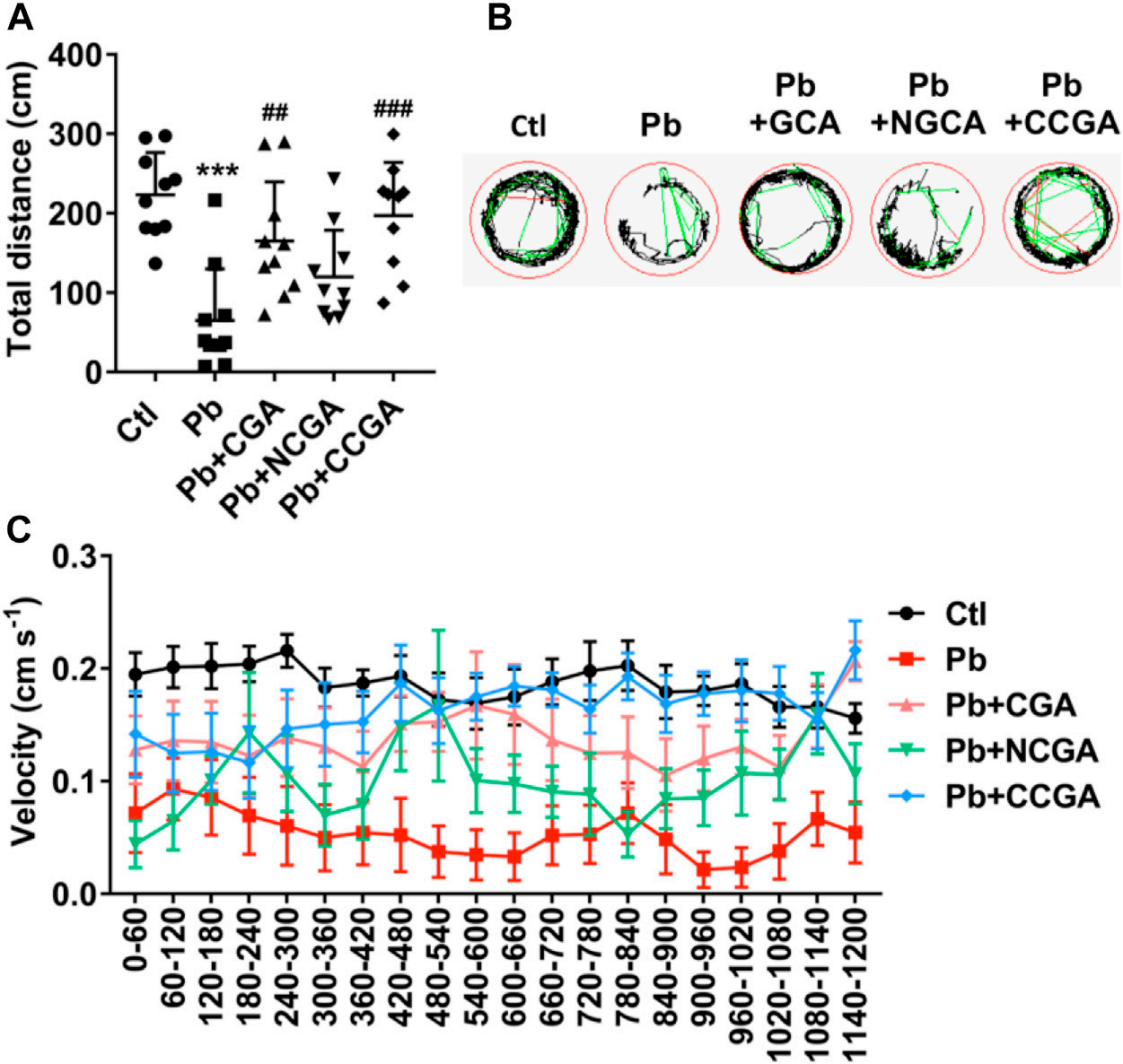
Swimming behavior test of zebrafish at 120 hpf. **(A)** Total distance traveled in 20 min (*n* = 10); ^***^ *p* < 0.001 vs. control and ^##^ *p* < 0.01, ^###^ *p* < 0.001 vs. Pb. **(B)** The digital tracking map. High-speed (v > 5 cm/s) movement is represented in red lines, medium-speed (2 cm/s < v < 5 cm/s) movement is depicted in green lines, and low-speed (v < 2 cm/s) movement is represented in black lines. (C) The swimming speed of zebrafish larvae with different treatment. Average speed in every 60 s was calculated.

To assess whether CGA, NCGA, and CCGA co-treatment ameliorated Pb-induced locomotor impairment, we have calculated the total distance traveled and swimming speed of the corresponding experimental groups. The swimming behavior test of zebrafish at 120 hpf demonstrates a significant decline (^***^ *p* < 0.001) in the total distance traveled in the Pb-administered group when compared to the control group (Figure 6A), suggesting a Pb-induced locomotor impairment. Co-treatment with CGA (^##^ *p* < 0.01) and CCGA (^###^ *p* < 0.001) significantly relieved Pb-induced locomotor impairment as evident by an increase in total distance traveled when compared to the Pb group (Figure 6A). Worth mentioning here is that NCGA does not significantly increase the total distance traveled in comparison to the Pb group.

The finding regarding total distance traveled is corroborated in digital tracking and mapping, where the high-speed, medium-speed, and low-speed movement of larvae is depicted in red lines, green lines, and black lines, respectively (Figure 6B).

In addition, the swimming speed of zebrafish showed a similar pattern with the total distance traveled. Co-treatment with CGA, NCGA, and CCGA increased the Pb-induced decline in swimming speed (Figure 6C), reflecting that CGA, NCGA, and CCGA possess protective effect on the locomotor pattern (Figure 6C).

### Effect on the Expression of Neurodevelopmental Genes

With an aim to investigate the impact of Pb on the genes related to neurodevelopment and protective effect of CGA, NCGA, and CCGA co-treatment against the same, we have studied the mRNA expression level of several neurodevelopmental genes (*c-fos*, *gfap*, *mbp*, *pparγ*, *tuba1b*, *bdnf*, and *dat*)*.*

**FIGURE 7:**
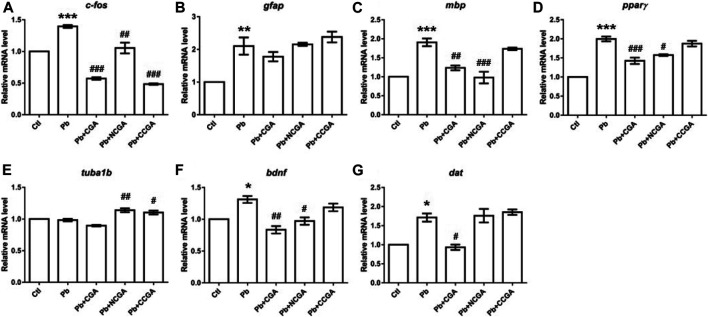
Transcription of genes related to neurodevelopment. The graph plot is represented as fold changes in the mRNA expression of c-fos **(A)**, gfap **(B)**, mbp **(C)**, pparγ **(D)**, tuba1b **(E)**, bdnf **(F)**, and dat **(G)**. Data were represented as mean ± SEM, n = 3, and statistically analyzed by one-way ANOVA followed by Dunnett’s multiple comparison test. ^*^ *p* < 0.05, ^**^ *p* < 0.01, ^***^ *p* < 0.001 vs. control and ^#^ *p* < 0.05, ^##^ *p* < 0.01, ^###^ *p* < 0.001 vs. Pb.

*c-fos* is a neurodevelopmental gene (Jin et al., 2019a) whose mRNA expression level was upregulated significantly upon Pb exposure (^***^ *p* < 0.001) when compared to the control group (Figure 7A). On the contrary, there is a significant downregulation in the expression of *c-fos* upon co-treatment with CGA (^###^ *p* < 0.001), NCGA (^##^ *p* < 0.01), CCGA (^###^ *p* < 0.001) in comparison to the Pb group (Figure 7A).

The mRNA expression of the neurodevelopmental gene glial fibrillary acidic protein (*gfap*) was increased significantly in the Pb group (^**^ *p* < 0.01) in comparison to that in the control group (Figure 7B). CGA nonsignificantly decreased the *gfap* mRNA expression, whereas NCGA did not modulate an expression of *gfap*, and CCGA nonsignificantly increased the *gfap* expression when compared to the Pb group (Figure 7B).

Myelin basic protein (*mbp*) is the gene associated with neurodevelopment and has been studied in zebrafish to investigate the gene transcription associated with neurodevelopment (Chen et al., 2020; Gu et al., 2020). Pb exposure led to a significant upregulation in the expression level of *mbp* (^***^ *p* < 0.001) when compared to that in the control group (Figure 7C). Co-treatment with CGA (^##^ *p* < 0.01) and NCGA (^###^ *p* < 0.001) significantly lowered the Pb-induced upregulated expression of *mbp*. However, no modulation in *mbp* expression was observed with CCGA co-treatment (Figure 7C).

Peroxisome proliferator–activated receptor-gamma (*pparγ*) mRNA expression was significantly increased upon Pb exposure (^***^ *p* < 0.001) which in turn was reduced upon co-treatment with CGA (^###^ *p* < 0.001) and NCGA (^#^ *p* < 0.05) (Figure 7D). However, CCGA did not modulate the expression of *pparγ* when compared to the Pb group (Figure 7D).

Tubulin alpha 1b (*tuba1b*) gene expression is crucial for CNS development and regeneration in zebrafish (Veldman et al., 2010). There was no modulation in mRNA expression of *tuba1b* in the Pb group when compared to the control group (Figure 7E). However, co-treatment with NCGA (^##^ *p* < 0.01) and CCGA (^#^ *p* < 0.05) significantly increased the expression of *tuba1b* when compared to the Pb group (Figure 7E), reflecting its role in CNS development.

Brain-derived neurotrophic factor (*bdnf*) expression is crucial for the developing brain in zebrafish (De Felice et al., 2014). When compared to the control group, we observed a significant upregulated mRNA expression of *bdnf* in the Pb group (^*^ *p* < 0.05), which was in turn reduced upon co-treatment with CGA (^##^ *p* < 0.01) and NCGA (^#^ *p* < 0.05) (Figure 7F).

Dopamine transporter (*dat*) is a membrane transport protein which stops the dopamine action by reuptake and has a role in embryonic development (Holzschuh et al., 2001). We observed an increased level of *dat* mRNA expression (^*^ *p* < 0.05) in the Pb group when compared to the control group (Figure 7G). The Pb-induced upregulated mRNA expression of *dat* was reduced by CGA (^#^ *p* < 0.05), whereas NCGA and CCGA did not modulate an expression of *dat* when compared to the Pb group (Figure 7G).

### Effect on the Expression of Genes Related to Oxidative Stress

Pb toxicity is correlated with inducing oxidative stress of organisms, leading to the inactivation of antioxidant enzymes (Zhang et al., 2014; Fan et al., 2020). Hence, to evaluate whether CGA, NCGA, and CCGA co-treatment ameliorated Pb-induced oxidative stress, we have assessed the mRNA expression of several genes related to oxidative stress (*sod2*, *sod1*, *cat*, *gclm*, *gsto2*, and *gpx4a*).

**FIGURE 8:**
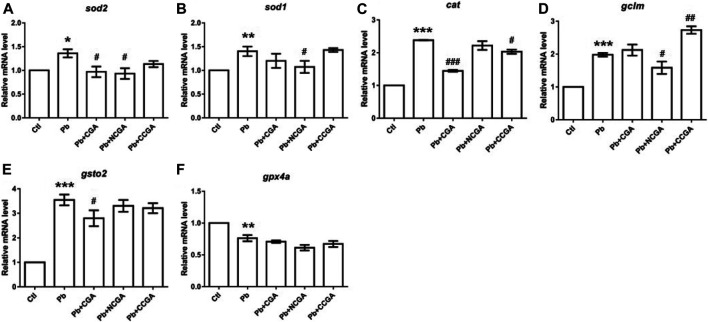
Transcription of genes related to oxidative stress. The graph plot is represented as fold changes in the mRNA expression of sod2 **(A)**, sod1 **(B)**, cat **(C)**, gclm **(D)**, gsto2 **(E)**, and gpx4a **(F)**. Data were represented as mean ± SEM, n = 3, and statistically analyzed by one-way ANOVA followed by Dunnett’s multiple comparison test. ^*^ *p* < 0.05, ^**^ *p* < 0.01, ^***^ *p* < 0.001 vs. control and ^#^ *p* < 0.05, ^##^ *p* < 0.01, ^###^ *p* < 0.001 vs. Pb.

We reported significant increase in mRNA expression of genes related to superoxide dismutase (sod) activity, that is, *sod2* (^*^ *p* < 0.05) (Figure 8A) and *sod1* (^**^ *p* < 0.01) (Figure 8B); and catalase (*cat*) activity (^***^ *p* < 0.001) (Figure 8C) in the Pb group in comparison to the control group. CGA (^#^ *p* < 0.05) and NCGA (^#^ *p* < 0.05) co-treatment significantly decreased Pb-induced increased mRNA expression of *sod2* (Figure 8A), whereas NCGA (^#^ *p* < 0.05) significantly reduced mRNA expression level of *sod1* (Figure 8B). In addition, CGA (^###^ *p* < 0.001) and CCGA (^#^ *p* < 0.05) co-treatment significantly downregulated Pb-induced upregulated mRNA expression of *cat*, whereas NCGA co-treatment did not significantly modulate mRNA expression of *cat* (Figure 8C).

There is a significant upregulation in the mRNA expression of antioxidant genes *gclm* (^***^ *p* < 0.001) (Figure 8D) and *gsto2* (^***^ *p* < 0.001) (Figure 8E), and downregulation in the expression of *gpx4a* (^**^ *p* < 0.01) (Figure 8F) in the Pb-exposed group when compared to the control group. NCGA co-treatment reduced (^#^ *p* < 0.05) *gclm* (Figure 8D) expression, whereas CCGA co-treatment increased (^##^ *p* < 0.01) the *gclm* expression (Figure 8D) when compared to the Pb-treated group. CGA (^#^ *p* < 0.05) treatment downregulated Pb-induced upregulated expression of *gsto2* (Figure 8E), whereas NCGA and CCGA did not modulate an expression of *gsto2* (Figure 8E) when compared to the Pb group. Worth mentioning here is that CGA, NCGA, and CCGA co-treatment did not significantly modulate mRNA expression of *gpx4a* when compared to the Pb group (Figure 8F).

Our findings support the notion that Pb led to impairment in oxidative stress, which was ultimately restored upon CGA, NCGA, and CCGA co-treatment.

### Effect on the Parkinsonian and Autophagy Related Genes

Autophagy is a cellular phenomenon that destroys cellular unfolded/misfolded proteins and damaged/unnecessary organelles (Liu et al., 2019). Cell autophagy has been reported as among the mechanism behind the Pb-induced neurotoxicity (Zhang et al., 2012; Liu et al., 2019). To investigate the protective role of autophagy in Pb-induced developmental neurotoxicity, we assessed an mRNA expression pattern of several autophagy-related genes which include Park7 (*dj1*), PTEN-induced putative kinase 1 (*pink1*), E3 ubiquitin protein ligase (*parkin*), autophagy/beclin-1 regulator 1a (*ambra1a*), unc-51–like autophagy-activating kinase 1 (*ulk1b*), unc-51–like autophagy-activating kinase 2 (*ulk2*), and autophagy-related gene 5 (*atg5*).

**FIGURE 9:**
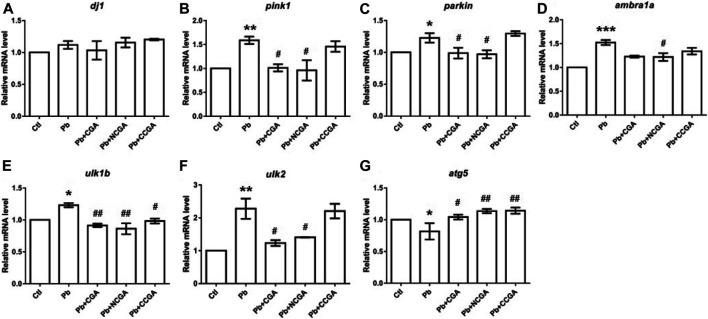
Transcription of genes related to parkinsonian and autophagy. The graph plot is represented as fold changes in the mRNA expression of dj1 **(A)**, pink1 **(B)**, parkin **(C)**, ambra1a **(D)**, ulk1b **(E)**, ulk2 **(F)**, and atg5 **(G)**. Data were represented as mean ± SEM, *n* = 3, and statistically analyzed by one-way ANOVA followed by Dunnett’s multiple comparison test. ^*^ *p* < 0.05, ^**^ *p* < 0.01, ^***^ *p* < 0.001 vs. control and ^#^ *p* < 0.05, ^##^ *p* < 0.01, ^###^ *p* < 0.001 vs. Pb.

There was a nonsignificant elevation in the mRNA expression of *dj1* in the Pb group when compared to the control group (Figure 9A). Surprisingly, co-treatment with CGA, NCGA, and CCGA did not exert any significant modulation in the mRNA expression level of *dj1* when compared to the Pb group (Figure 9A).

**FIGURE 10:**
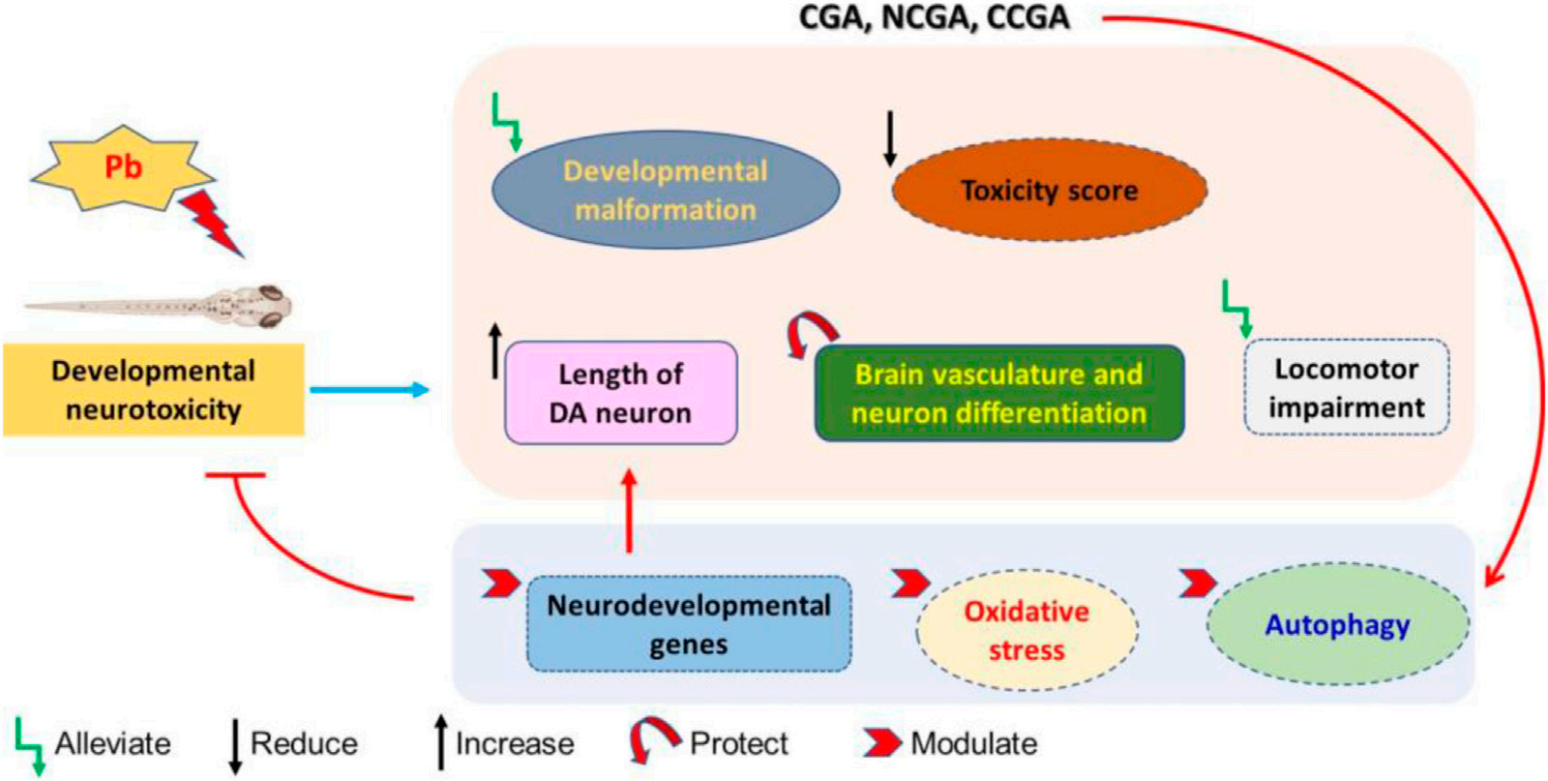
Schematic representation of protective effect of CGA, NCGA, and CCGA against Pb-induced developmental neurotoxicity co-treatment of CGA, NCGA, and CCGA alleviated developmental malformation, reduced toxicity score, increased the length of DA neuron region, protected brain vasculature and neuron differentiation in the CNS, ameliorated locomotor impairment, modulated neurodevelopmental genes (*c-fos*, *gfap*, *mbp*, *pparγ*, *tuba1b*, *bdnf*, and *dat*), oxidative stress-related genes (*sod2*, *sod1*, *cat*, *gclm*, *gsto2*, and *gpx4a*), and parkinsonian and autophagy-related genes (*dj1*, *pink1*, *parkin*, *ambra1a*, *ulk1b*, *ulk2*, and *atg5*). Summing up, our study demonstrates that co-treatment with CGA and its analogues NCGA and CCGA protects against Pb-induced developmental neurotoxicity. The protective mechanism of CGA, NCGA, and CCGA co-treatment might not be only due to the inhibition of apoptosis also protection of brain vasculature but also due to the inhibition of Pb exposure induced oxidative stress and autophagy in zebrafish (Figure 10). Our results provide proof of concept that CGA, NCGA, and CCGA might represent the future treatment against Pb poisoning.

In comparison to the control group, there was a significant elevation in the mRNA expression of *pink1* (^**^ *p* < 0.01) (Figure 9B), *parkin* (^*^ *p* < 0.05) (Figure 9C), *ambra1a* (^***^ *p* < 0.001) (Figure 9D), *ulk1b* (^*^ *p* < 0.05) (Figure 9E), and *ulk2* (^**^ *p* < 0.01) (Figure 9F) in the Pb group. The co-treatment with CGA (^#^ *p* < 0.05) and NCGA (^#^ *p* < 0.05) significantly reduced the mRNA expression of *pink1* (Figure 9B) and *parkin* (Figure 9C), respectively, whereas CCGA did not exert any significant modulation on *pink1* and the *parkin* level. In comparison to the Pb group, NCGA significantly reduced (^#^ *p* < 0.05) the expression of *ambra1a* (Figure 9D), whereas CGA and CCGA exerted significant modulation in the same (Figure 9D). In addition, CGA (^##^ *p* < 0.01), NCGA (^##^ *p* < 0.01), and CCGA (^#^ *p* < 0.05) co-treatment significantly downregulated mRNA expression of *ulk1b* in comparison to the Pb group (Figure 9E). Co-treatment with CGA (^#^ *p* < 0.05) and NCGA (^#^ *p* < 0.05) decreased Pb-induced increased mRNA expression of *ulk2* when compared to the Pb group (Figure 9F), whereas CCGA did not cause significant modulation in the level of *ulk2* (Figure 9F).

When compared to the control group, Pb led to the significant reduction in mRNA expression of *atg5* (^*^ *p* < 0.05) (Figure 9G), which in turn was upregulated with CGA (^#^ *p* < 0.05), NCGA (^##^ *p* < 0.01), and CCGA co-treatment (^##^ *p* < 0.01) (Figure 9G).

All our findings reflect that Pb led to impairment in autophagy, which was in turn ameliorated upon co-treatment with CGA, NCGA, and CCGA.

## Discussion

Pb intoxication impacts the CNS, leading to structural disorders and behavioral impairment in several animal species (Müller et al., 2008; Liu et al., 2019). Despite the well-known phenomena of Pb-induced neurotoxicity, studies on the developmental neurotoxicity induced by Pb in zebrafish are very limited. The aim of this study was to study the impact of Pb exposure on neurodevelopment in zebrafish and to evaluate the protective effect of phenolic compounds CGA, NCGA, and CCGA against the Pb-induced developmental neurotoxicity.

An earlier study has reported that the natural phenylpropene, *α*-asarone, did not significantly affect mortality and hatching rate, whereas there was an increased malformation rate in zebrafish in a concentration-dependent pattern (Shang et al., 2020). In a similar line, we observed that Pb-exposed, and CGA, NCGA, and CCGA co-treated group exerted no effect on the hatching rate and mortality when compared with the control group. In addition, the corresponding experimental group exerted no significant difference in morphology of zebrafish visualized at 48 and 72 hpf.

The study evaluating a toxicity score of nanoparticle toxicity in zebrafish reported a scoring spectrum for assessing the toxicity *via* screening the level of zebrafish malformation where the toxicity score ranges from score 0 to score 4 (Liu et al., 2012). Corroborating the findings from the said study, at 120 hpf, we observed that Pb-induced malformation such as PE, YM, BS, SE, TM, SG, and AB to be reflecting toxicity induced by Pb. Furthermore, the average toxicity score and malformation rate were higher in the Pb group, which were ameliorated upon co-treatment with CGA, NCGA, and CCGA. This finding suggests that CGA and its analogues might protect against the Pb-induced developmental neurotoxicity.

Apoptosis is a regulated phenomenon in which cell death is executed to retain the steady state under physiological circumstances and for responding to several stimuli (Xu et al., 2006). An ample amount of findings suggest that Pb does induce apoptosis in several cell types (Yedjou et al., 2010; Baranowska-Bosiacka et al., 2013; Yedjou et al., 2016; Tu et al., 2018). TUNEL staining is useful for detecting apoptosis in zebrafish larvae (Zhang S. et al., 2019; Gu et al., 2020). Herein, we observed a significant reduction in the length of DA neurons upon Pb exposure, which was ultimately rescued upon CAG, NCGA, and CCGA co-treatment. This finding supports the notion that Pb exposure leads to apoptosis during neurodevelopment in zebrafish and CGA, and its analogues can ameliorate Pb-induced apoptosis.

Although the mechanism behind Pb-induced neurotoxicity is not yet fully understood, there is an understanding that post-inhalation or ingestion, Pb enters the bloodstream and penetrates the brain *via* both the blood–brain barrier (BBB) and the blood–cerebrospinal fluid (CSF) barrier. In turn, the endothelial cells present on the BBB microvasculature and the choroid plexus cells that consist of the blood–CSF barrier accumulate Pb, resulting in leaky barriers (Caito and Aschner, 2017). Hence, any potential compound tested against Pb-induced developmental neurotoxicity should be able to protect the neuron differentiation in the CNS and the brain vasculature. In our current study, Pb exposure led to disrupted neuron differentiation in the CNS and significant loss of brain vasculature as evidenced by the decrease in the fluorescence of the differentiated CNS neuron region and reduction in the number of blood vessels in the brain, respectively. All these Pb-induced modulation were significantly ameliorated by CGA, NCGA, and CCGA co-treatment, reflecting the potential to protect against Pb-induced disruption in differentiated neurons and brain vasculature.

During the early developmental stage, locomotor behavior is acknowledged as a crucial neurodevelopment index and is enormously susceptible to environmental contaminants (Rihel et al., 2010). Reduction in the swimming speed in zebrafish larvae was observed upon exposure to pentabromobenzene and hexabromobenzene as an indicator of their neurotoxicity (Chen et al., 2020). In a similar pattern, in our study, Pb exposure led to locomotor impairment as evidenced by a reduction in the total distance traveled and a decrease in swimming speed by the zebrafish. On the contrary, Pb-induced locomotor impairment was ameliorated by co-treatment with CGA, NCGA, and CCGA as evidenced by an increase in the total distance traveled and increase in swimming, reflecting that CGA and its analogues protect against Pb-induced developmental neurotoxicity.

Although the Pb-induced neurotoxicity is an extensively reported toxicity phenomenon, the impacts of Pb exposure on neurodevelopment, oxidative stress, and autophagy are scarce in the literature. It is well reported that *c-fos* is a marker of neuronal activation and upregulation of *gfap* denotes neurotoxicity and is present in the nervous system of zebrafish (Herrera and Robertson, 1996; Ingham and McMahon, 2001; Fan et al., 2010). A marker of myelination, *mbp*, is abundantly expressed in the nervous system in the developmental stages of zebrafish embryos (Brösamle and Halpern, 2002), whereas *tuba1b* exerts a crucial role in the CNS development (Veldman et al., 2010). We found that Pb exerted disturbing effect on the expression patterns of key genes regulating neurodevelopment such as *c-fos*, *gfap*, *mbp*, *pparγ*, *tuba1b*, *bdnf*, and *dat*. Therefore, the upregulation of these gene expressions plausibly reflects disruptive effect on the structure and function of CNS *via* influencing the formation of cytoskeleton, axon growth, synaptogenesis, and neuronal differentiation, resulting in developmental neurotoxicity. All these Pb-induced modulation in genes transcription related to neurodevelopment were in turn modulated upon CGA, NCGA, and CCGA co-treatment, reflecting the potential against developmental neurotoxicity induced by Pb.

Oxidative stress is simply acknowledged as an imbalance in the prooxidants and antioxidants that are responsible for oxidative damage. The antioxidant defense mechanism, together with the antioxidant enzymes, is crucial in preserving the redox state and defending against oxidative damage (Gu et al., 2020). Studies have reported that Pb-induced toxicity is related to oxidative stress due to overproduction of ROS (Chen et al., 2003), reduction of reduced glutathione (GSH) (Jozefczak et al., 2012), and interference of activities of antioxidant enzymes such as SOD (Vaziri et al., 2003). The expression level of several genes including *sod2*, *sod1*, *cat*, *gclm*, *gsto2*, and *gpx41* related to oxidative stress after Pb exposure reflects the oxidative damage which was ultimately ameliorated by CGA, NCGA, and CCGA co-treatment, reflecting its antioxidant defense mechanism.

Wealthy amount of evidence has reported the possibility of Pb-induced neurodegeneration, leading to neurodegenerative disorders (Han et al., 2007; Pachauri et al., 2009; Zizza et al., 2013; Zhou et al., 2020); however, the underlying mechanism behind Pb-induced neurodegeneration needs to be further elucidated. Hence, we aimed to unravel whether Pb possesses risk of causing neurodegenerative diseases in the zebrafish model and assessed the modulation in the transcriptional level of autophagy-related and parkinsonian genes in the brain. This will ascertain the risk of Pb in inducing neurodegenerative diseases *in vivo* and to precisely understand the role of autophagy in this process. We observed that the mRNA expression of all the parkinsonian and autophagy-related genes (*dj1*, *pink1*, *parkin*, *ambra1a*, *ulk1b*, and *ulk2*) was increased, but *atg5* expression was decreased, suggesting that Pb exposure plausibly triggered autophagy that plays key roles in parkinsonism. Our findings were in corroboration with those in an earlier study that reported transcriptional alterations of parkinsonian and autophagy-related genes involved in neurotoxicity induced by nanomaterial exposure in zebrafish (Jin et al., 2021). Our study explored the behavioral toxicity of Pb and unraveled the association among Pb exposure, neurodegeneration, and the alteration of autophagy levels in this process.

## Data Availability

The raw data supporting the conclusions of this article will be made available by the authors, without undue reservation.
