# Supplementary material for: Protective Effect of Chlorogenic Acid and Its Analogues on Lead-Induced Developmental Neurotoxicity Through Modulating Oxidative Stress and Autophagy
Source: Front Mol Biosci. 2021 Jun 11;8:655549. doi: 10.3389/fmolb.2021.655549 (PMC8226318; doi:10.3389/fmolb.2021.655549)
Supplement: Supplementary file 1 [file Table1.DOCX]

**SUPPLEMENTARY INFORMATION**

**Protective Effect of Chlorogenic Acid and Its Analogues on Lead-Induced Developmental Neurotoxicity through Modulating Oxidative Stress and Autophagy**

Xiuna Ji ^1,2^**^#^**, Baokun Wang ^1,2#^, Yam Nath Paudel ^3^**^#^**, Zhihui Li ^4^, Shanshan Zhang ^1,2^, Lei Mou ^1,2^, Kechun Liu ^1,2^, and Meng Jin ^1,2^*****

^1^Biology Institute, Qilu University of Technology (Shandong Academy of Sciences), 28789 East Jingshi Road, Ji’nan 250103, Shandong Province, People’s Republic of China

^2^Engineering Research Center of Zebrafish Models for Human Diseases and Drug Screening of Shandong Province, 28789 East Jingshi Road, Ji’nan 250103, Shandong Province, People’s Republic of China

^3^Neuropharmacology Research Strength, Jeffrey Cheah School of Medicine and Health Sciences, Monash University Malaysia, Bandar Sunway, Selangor, Malaysia

^4^ School of Bioengineering, Qilu University of Technology (Shandong Academy of Sciences), 3501 Daxue Road, Ji’nan 250353, Shandong Province, People’s Republic of China

***Corresponding Author**

Meng Jin, PhD

Biology Institute, Qilu University of Technology (Shandong Academy of Sciences),

28789 East Jingshi Road, Ji’nan 250103,

Shandong Province,

People’s Republic of China

Email: mjin1985@hotmail.com

Contact No: +86-13127104334

**Primers used for the gene expression**

| **S.N.** | **Gene** | **Primer** |
| --- | --- | --- |
| 1 | *c-fos* | F: TGCAGCACGGCTTCACCGAG  R: CGGGCATGAAGAGATCGCCGT |
| 2 | *gfap* | F: GGATGCAGCCAATCGTAAT  R: TTCCAGGTCACAGGTCAG |
| 3 | *mbp* | F: AATCAGCAGGTTCTTCGGAGGAGA  R: AAGAAATGCACGACAGGGTTGACG |
| 4 | *pparγ* | F: TCTCCGCTGATATGGTGGAC  R: GTCGATGCCTGATATGCTGC |
| 5 | *tuba1b* | F: AATCACCAATGCTTGCTTCGAGCC  R: TTCACGTCTTTGGGTACCACG |
| 6 | *bdnf* | F: AACTCCAAAGGATCCGCTCA  R: GCAGCTCTCATGCAACTGAA |
| 7 | *dat* | F: CTAATCGCCTTCTCCAGCTACA  R: GGCCACGTTGTGTTTCTGTGACAT |
| 8 | *sod2* | F: TGTTGGTTGGTCGCTTGTAT  R: GTGCTTCTGTCTGGAGGTCA |
| 9 | *sod1* | F: AACATGGTTTCCACGTCCAT  R: CGGTCACATTACCCAGGTCT |
| 10 | *cat* | F: AGGGCAACTGGGATCTTACA  R: GATCCTTCAGGTGAGTCTGC |
| 11 | *gclm* | F: ATCCATCAGAAGTGCGGTAG  R: TGCAGGTGTGTCAGTGTCT |
| 12 | *gsto2* | F: ATGGCTTCATCTCCAAAATGC  R: AGGGCAGAATCTCATGCTGTAG |
| 13 | *gpx4a* | F: CAGGAACCAGGAACTAATTCCCAAATC  R: TCCAAGGAATCCTTTTCCATTAGGCT |
| 14 | *dj1* | F: TGTTACTGTCGCAGGTCTGG  R: CAGGCAGAAGAACAACGTCA |
| 15 | *pink1* | F: GGCAATGAAGATGATGTGGAAC  R: ATCACGTTGGGATGAGCACT |
| 16 | *parkin* | F: GCGAGTGTGTCTGAGCTGAA  R: GCCCTGAAGTGTGGATTCAT |
| 17 | *ambra1a* | F: TAACCAGGAAACTGGCCAAC  R: AATATGCTGCAGGGGACAAC |
| 18 | *ulk1b* | F: AGGCCGAAAGTCTCACTTCA  R: AGCCATGTACATCGGAGACC |
| 19 | *ulk2* | F: ACCTCTGATTGGCTGACAAAAT  R: GAGATTGCAAGAGGCTTGAGTT |
| 20 | *atg5* | F: AGGGGATAACAGCACAAACG  R: CTTCTTATGCAGCGTGTCCA |
